# Combinatorial and rational synthesis of complex, base-modified aptamer libraries on microarrays

**DOI:** 10.1039/d6ra01942k

**Published:** 2026-05-19

**Authors:** Erika Schaudy, Jory Lietard

**Affiliations:** a Institute of Inorganic Chemistry, University of Vienna Vienna 1090 Austria erika.schaudy@univie.ac.at

## Abstract

Chemically modifying the backbone, sugar, or nucleobase moieties of nucleic acids greatly expands their functional repertoire. Nucleobase modifications have received particular attention for their proven ability to generate a greater diversity of intra- and intermolecular interactions. This broader interaction landscape has facilitated advances in the identification of aptamers with high affinity to different target molecules, including proteins. As common practice, SELEX (Systematic Evolution of Ligands by EXponential enrichment) is used to select the best binders from a large pool of random oligonucleotides. However, the combinatorial space of SELEX is usually limited to *n* = 4 different nucleotides and it does not account for position-specific effects of target binding. In contrast, photolithographic synthesis of oligonucleotide microarrays allows for (a) full control of the position of a modified nucleotide in a sequence, (b) the implementation of multiple nucleic acid building blocks in addition to the four canonical nucleotides, and (c) the chemical synthesis of hundreds of thousands of unique sequence variants in parallel and on the same surface. In this work, we explored photolithographic microarray synthesis with two uracil analogs carrying the side chain of the amino acids serine and tyrosine on position C5 (dU-Ser, dU-Tyr). Hybridization experiments showed an enhanced duplex stability of homopolymer strands of these modified nucleotides compared both to dU and dT. Additionally, we synthesized permutation libraries of two known streptavidin aptamers by substituting the dT by dU, dT, dU-Ser and dU-Tyr in all possible combinations. In binding assays, we observed positions at which the presence of modified dUs increases protein binding, and locate other sites that do not tolerate chemical modifications. In summary, this project highlights the power of oligonucleotide microarrays in studying affinity patterns in aptamers and demonstrates the value of incorporating base-modified nucleotides in photolithographic synthesis.

## Abstract 2

Complementing photolithographic microarray synthesis with nucleobase-modified DNA nucleotides yields large oligonucleotide libraries and allows for full control in combining type and position of modifications along any sequence.

## Graphical abstract

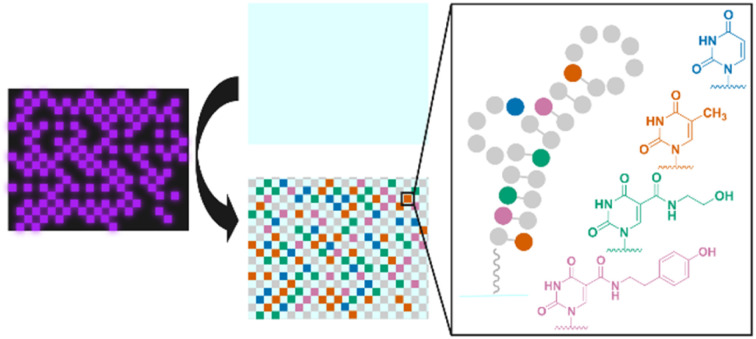

## Introduction

Nucleic acids are not only media for the storage and transmission of genetic information, but also highly versatile biopolymers capable of performing diverse biological functions. Beyond encoding proteins, they can fold into complex three-dimensional structures that enable catalysis, molecular recognition, and regulation.^1^ However, the chemical and functional diversity of natural nucleotides is inherently limited to a four-letter chemical space, constraining the range of structures, binding interactions, and physicochemical properties that can be explored. This functional repertoire has been expanded *via* the introduction of chemical modifications to the phosphodiester backbone or the sugar moiety, enabling targeted fine-tuning of properties such as stability, affinity, and nuclease resistance, particularly relevant in the field of oligonucleotide therapeutics.^2^ However, a much broader functional space has been uncovered with nucleobase modifications: nucleobases augmented with functional groups of varying size,^3^ charge,^4^ redox-activity^5,6^ and hydrophobicity^7^ or even entirely artificial nucleobases^8–10^ have also been developed. These efforts aim to broaden the spectrum of intra- and intermolecular interactions to ultimately unlock the full potential of nucleic acids in applications requiring high specificity and affinity to other molecules. This is of particular interest in aptamer research, where the potential to chemically diversify the functional repertoire of oligonucleotides has been employed as a means to overcome the limitations of canonical nucleic acids,^11^*i.e.*, unmodified aptamers preferentially bind positively charged and structurally defined targets by hydrogen bonding and electrostatic contacts,^12^ whereas hydrophobic, neutral, or featureless surfaces remain challenging. Different nucleobase modifications have been described to improve our ability to address such difficult targets.^11^ For example, an aptamer with cubane-modified dU has been described for its ability to form hydrophobic interactions, but also an unusual type of hydrogen bonding, allowing to distinguish between closely related malaria biomarkers,^13^ and the implementation of large nucleobase modifications can improve the affinity of aptamers targeting small molecules by increasing the number of functional groups potentially involved in intra- and intermolecular interactions.^14^ Inspired by the functional variety brought about by amino acids in proteins, the introduction of new functional groups in nucleic acid constructs that can modulate site-specific hydrophobicity^15^ has opened up novel modes of interactions with target molecules,^16,17^ resulting in the identification of DNA aptamers for several clinically relevant proteins which were not addressable by unmodified nucleic acids^18^ and leading to the commercialization of so-called SOMAmers (Slow Off-rate Modified Aptamers).

**Fig. 1:**
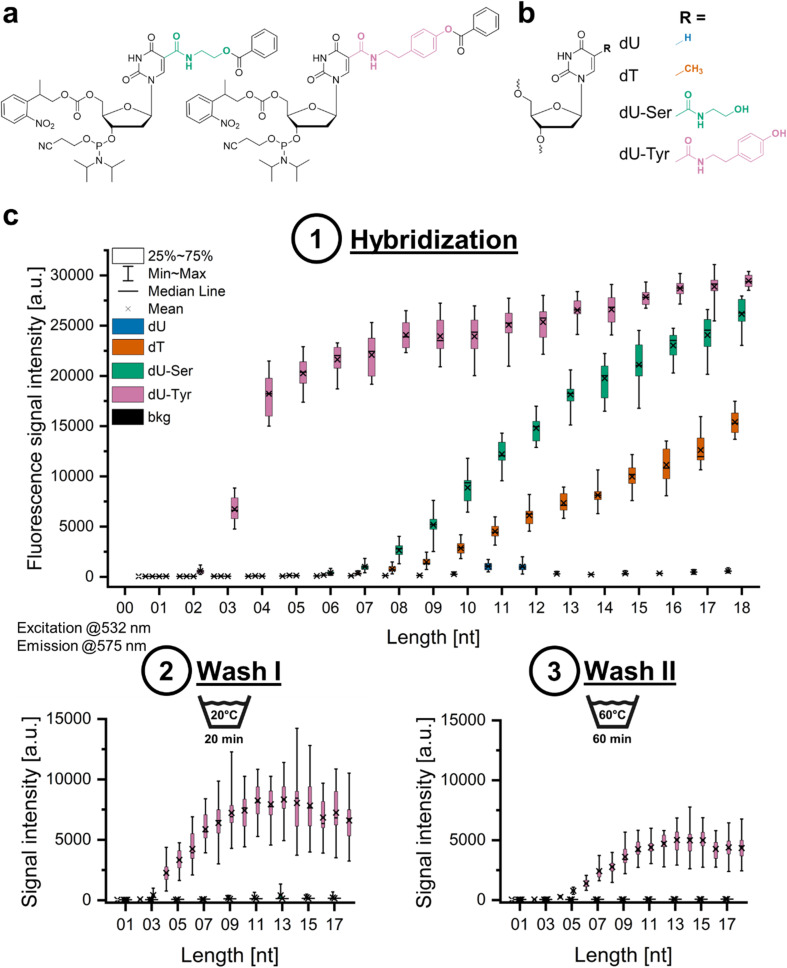
Overview on the nucleosides investigated in photolithographic microarray synthesis and their behaviour in hybridization and wash-off experiments. (a) Structures of the dU-Ser and dU-Tyr phosphoramidites with a 5′ NPPOC photosensitive protecting group. (b) Structures of the nucleosides compared in this study. (c) Fluorescence signal intensities recorded (1) upon hybridization of a dA_20_-Cy3 probe to strands of dU (data shown in blue), dT (orange), dU-Ser (green) and dU-Tyr (pink) with a length of 1–18 nt in comparison to the background signal (0 nt, black); (2) after washing the microarray in water for 20 min; (3) after washing for 60 min in 60 °C water.

The identification of aptamers typically relies on a selection pressure imposed on a large pool of oligonucleotides with random sequences to identify the candidates with highest affinity to a target, a process first described in 1990 and since known as SELEX (Systematic Evolution of Ligands by EXponential enrichment).^19^ While this method can screen extremely complex sequence libraries, it heavily relies on the use of polymerases in the process, limiting chemical diversity in nucleic acids to what these enzymes are willing to accept as substrates. Though enzyme engineering efforts have yielded polymerases that show a more relaxed attitude towards non-natural nucleotide triphosphates,^20^ nucleotide modifications can still significantly affect their catalytic efficiency,^21^ requiring individual assessment of the substrate's compatibility with the respective polymerase.^22^ Additionally, the typical approach to introduce modified nucleotides in SELEX is to fully exchange an unmodified dNTP for its base-modified version,^23^ which may have adverse effects on target binding. Due to this bulk substitution, the method has only limited capacity to address the position-specific impact of nucleobase modifications within aptamer structures.^24,25^ A different strategy has recently been described that can control the location of noncanonical nucleotides using templated ligation of nucleobase-modified codons.^26^ An alternative to enrich the combinational space of DNA in a non-enzymatic approach is the introduction of base-modified nucleotides during oligonucleotide solid-phase synthesis.^27^ This chemical synthesis approach is amenable to modifications beyond simple nucleic acids, as was shown with the insertion of amino acid side chains on a thrombin aptamer in the form of amino acid–nucleic acid hybrids (ANHs).^28^ Therefore, ANHs represent an appealing alternative for the site-specific introduction of amino acid functionalities into aptamers in a chemical synthesis approach. But solid-phase synthesis is inherently low throughput and only delivers a few variants per run, severely limiting the number of combinations that can be tested in a reasonable amount of time. A multiplexed approach to oligonucleotide synthesis overcomes the throughput barrier, one example of multiplexing being spatially selective photodeprotection. This process, called photolithography, yields nucleic acid microarrays^29^ that can accommodate a large variety of chemical modifications.^30–35^ In this work, we enrich the chemical toolbox for photolithographic synthesis to DNA libraries supplemented with base-modified nucleotides. Following the example set by the SOMAmer approach^36^ and other recent reports,^37^ we investigated deoxy-uridine decorated with functional groups featuring structural similarity with two amino acids, serine and tyrosine (dU-Ser and dU-Tyr), which have been described as key contributors to specific protein binding in antibody paratopes^38,39^ and engineered monobodies.^40^ We decided to adopt the attachment strategy of linking the functional group of the amino acid side chain *via* an amide bond to the C5 position of the pyrimidine base (see Fig. 1a). We expect this amide functionality to be unreactive in photolithographic synthesis conditions but to potentially participate in interactions with target molecules in the context of aptamer binding.^17^ Hybridization experiments on these chimeric microarrays suggest a high efficiency of the two novel phosphoramidites in the context of photolithographic synthesis. We also investigate the impact of combinations of dU variants (dU, dT, dU-Ser, dU-Tyr) in two different streptavidin aptamers on target binding. The microarray platform allows us to investigate the combinatorial effect of modifications with full control of the exact position, an approach that differs from the selection workflow of SOMAmers *via* SELEX, while addressing many variants in parallel. On-surface evaluation of these large permutation libraries indicates that target affinity is dependent both on the type of modification and the position in the sequence. To the best of our knowledge, no other currently available method would allow for the discovery of these motifs with mixed nucleotide modifications that either improve or deteriorate protein binding affinity.

## Experimental

### Microarray synthesis

The principle of maskless array synthesis (MAS) has been extensively discussed elsewhere.^41–43^ Briefly, the synthesis relies on the interplay of an optical setup and an oligonucleotide synthesizer. The optical system directs 365 nm UV light from a UV-LED (Nichia NVSU333A) onto a 0.7″ XGA DMD (Texas Instruments). The tilt of the micromirrors of the DMD is controlled by masks (1-bit image files), containing the information which mirrors should be tilted to “off” (dark spot in image, UV light reflected away) or “on” position (bright spot in image, UV light directed into the reaction chamber). The reaction chamber carries two glass microscope slides (Schott Glass D) that have been functionalized with *N*-(3-triethoxysilylpropyl)-4-hydroxybutyramide (abcr AB129323) as substrates for synthesis, is positioned in the focal plane of the optical setup and is connected to the oligonucleotide synthesizer (Expedite 8909, PerSeptive Biosystems). The synthesizer pumps the reagents between the two glass slides in reaction cycles synchronized with the optical setup to ensure the presence of the correct reagent during light exposure. Reagents for synthesis were purchased at Sigma (oxidizer L060080, DCI activator L032080), exposure solvent was prepared as 1% imidazole in DMSO (Sigma 56750 and 34943) and acetonitrile was supplied from Thermo Fisher Scientific. CAPS (Cap B L050080 & Fast Deprotection Cap A L070000 from Sigma) were used in the synthesis of all sequences except the linker for all microarrays that contain dG in order to prevent branching.^44^ Cyanoethyl-phosphoramidites with 5′-NPPOC protecting group (dU, dU-Ser, dU-Tyr – structures shown in Fig. 1a, custom-synthesized by ChemGenes following published procedures^45,46^) were coupled for 60 seconds and a radiant exposure of 8.6 J cm^−2^ (365 nm) was applied for photodeprotection. 5′-BzNPPOC protected monomers (Orgentis) were coupled for 15 seconds as described previously.^47^ A linker (C_10_ in hybridization experiments, T_20_ in aptamer experiments) was synthesized at the 3′-end of all interrogated sequences, background features contain only the linker sequence. Cyanoethyl and base protecting groups were removed after synthesis in a solution of ethylenediamine and ethanol (1 : 1, both from VWR) for 2 hours at room temperature.

### Coupling efficiency

The stepwise coupling efficiency of phosphoramidites was determined in an approach previously described in detail.^32^ Briefly, 1–12 monomers were consecutively coupled for either 15 s or 60 s, and each of these coupling events was followed by capping of unreacted hydroxyl groups. Cy3 phosphoramidite (Biosearch Technologies; 25 mM in ACN, 2 × 5 min coupling) was coupled in the last step of the synthesis, and the slides were washed in ACN for 2 h, treated for 2 h in a solution of EDA with ethanol (1 : 1), washed thoroughly with water and dipped into 0.1× SSC buffer before drying and scanning. Coupling failures result in capped strands that will be blocked from receiving Cy3, therefore, poor coupling efficiency will cause the accumulation of capped strands and result in decrease of fluorescent signal intensity. The median signal intensities were fit to an exponential decay model *y* = *ae*^−*bx*^ to calculate the stepwise coupling efficiency as the inverse of the decay rate *b*.

### Photolysis efficiency

The photolysis efficiency of the dU-Ser and dU-Tyr phosphoramidites described herein was assessed in direct comparison to dU monomers in a terminal labelling approach: after the coupling of a single nucleotide and capping, a UV light gradient covering the range from 0 to 13.5 J cm^−2^ was applied by exposing different features on the surface for a set amount of time, followed by coupling of Cy3 phosphoramidite (Biosearch Technologies; 50 mM in ACN, 2 × 5 min coupling). After synthesis, the slides were washed in ACN for 2 h, treated for another 2 h in a solution of EDA with ethanol (1 : 1), washed thoroughly with water and dipped into 0.1× SSC buffer before drying and scanning.

### Hybridization

Hybridization was performed in self-adhesive hybridization chambers (Grace Biolabs SA200) with 8.9 nM Cy3-labeled probe (for hybridization to strands composed of dU variants: DNA probe 5′-Cy3-GAAAAAAAAAAAAAAAAAAAA from Eurogentec, RNA probe 5′-Cy3-GDDDDrArArArArArArArArArArArArArArArArArArA from IDT; hybridization to mixed-base 25mer: DNA probe 5′-Cy3-GACCAGGGTGGTTCATGATGATGAC from Eurogentec) in MES buffer (100 mM MES, 1 M Na^+^, 20 mM EDTA, 0.01% Tween20) supplemented with 0.44 mg mL^−1^ acetylated BSA (Promega R3961) for 2 hours either at room temperature (DNA probe) or 4 °C (RNA probe). Arrays were washed with three buffers with decreasing salt concentration – first for 2 min in non-stringent wash buffer (0.9 M NaCl, 0.06 M sodium phosphate, 6 mM EDTA, 0.01% Tween20), followed by 1 min in stringent wash buffer (100 mM MES, 0.1 M NaCl, 0.01% Tween20), and for 10 s in final wash buffer (0.1× SSC) – followed by drying in a microarray centrifuge and scanning. Water washes were performed by shaking the microarray in a Falcon tube with ∼40 mL Milli-Q water for the given time at the indicated temperature.

### Aptamer experiments

#### Microarray design

Data shown for the binding of Cy3-labeled streptavidin are based on the aptamers St-2-1 (5′-A**TT**GAC̲C̲G̲C̲**T̲**G**T**G**T**G̲A̲C̲G̲C̲A̲ACAC**T**CAA**T**-3′–7 dTs in the native structure that were permuted to either dU, dT, dU-Ser or dU-Tyr are shown in bold, nucleotides in the predicted bulge and loop structures are underlined) and St-23-2 (5′-GAGGA**T**AA**T̲**C̲G̲C̲**T̲**CACCG̲A̲C̲G̲C̲A̲GG**T**G**TT**A**T**CTTC-3′).^48^ A reverse sequence with identical length and nucleotide composition as the St-2-1 aptamer (St-2-1_rev: 5′-TAACTCACAACGCAGTGTGTCGCCAGTTA-3′) was used to assess unspecific binding. The number of replicates for each sequence is limited by the dimensions of the microarray, feature size and the total number of variants to investigate. Using a 1 : 4 layout (1-mirror feature size within a square of 2 × 2 mirrors), the number of replicates per microarray varies between 10 and 15. Digital masks for microarray synthesis were generated using a custom-made MATLAB script.^41^

#### Aptamer binding assays

Aptamers were folded in binding buffer (50 mM Tris–HCl pH 7.6, 100 mM NaCl, 2 mM MgCl_2_, 1 mM CaCl_2_, 5 mM KCl) by incubating the microarray for 10 min on a thermomixer set to 95 °C, placing the slide on ice for 5 min followed by incubation at room temperature and slow rotation for 20 min. Cy3-labeled streptavidin (Jackson Immuno Research 016-160-084) served as target in aptamer assays, which were performed for 1 hour in binding buffer at room temperature with slow rotation. The target solution was pushed out of the reaction chamber by applying one chamber volume of binding buffer, emptying the chamber and rinsing once more with buffer. The reaction chamber was removed and the slides washed in 30 mL 1× binding buffer for 10 s, dried in a microarray centrifuge and scanned at 2.5 µm resolution on a GenePix microarray scanner (Molecular Devices) with laser and emission filter set for the detection of Cy3. Since water washes were insufficient to remove fluorescent signal from the slide and in order to avoid DNA damage due to harsh treatment conditions, proteinase K was applied for successfully setting back the signal on the microarray to background level again when needed.

#### Aptamer binding affinity

For investigation of aptamer binding affinity, a single microarray was treated as described above consecutively with increasing concentrations of streptavidin-Cy3 in binding buffer, ranging from 1 nM to 1.5 µM. Since the streptavidin stock provided by the supplier contains BSA as a protein stabilizer, BSA was supplemented to yield a concentration of 0.67 mg mL^−1^ independent of target protein concentration. The raw fluorescence signal intensity detected in each feature was corrected with background signal measured at the respective streptavidin concentration. The corrected data were analyzed using a Python script that fits a hyperbolic binding model to the median signal of each sequence variant. In order to account for noise in fluorescence data observed upon incubation with higher concentrations of streptavidin in particular in the low intensity range, only data points exceeding a signal intensity threshold of 10× SD of the background signal at highest target concentration and fits with *r*^2^ > 0.7 were retained.

### Data extraction and analysis

Cy3 fluorescence signal was detected using a GenePix microarray scanner (Molecular Devices) at 2.5 µm resolution using a 532 nm laser for excitation and a 575 nm filter. NimbleScan 2.1.68 (NimbleGen Systems Inc.) was used to align the scans with the corresponding design file (generated by MATLAB upon creation of the files required for photolithographic synthesis) and extract the fluorescence intensity data. Raw intensity data were manually cleaned up from outliers with inordinate high signal which typically can be attributed to surface artefacts (*e.g.* particles, salt crystals). Cleaned data were further processed in Python and Microsoft Excel. Python codes for data analysis and generation of sequence logos were developed with AI assistance. Graphs were generated with OriginPro 2025.

## Results and discussion

### Identification of synthesis parameters

We aimed to incorporate two custom-made photosensitive phosphoramidites, dU-Ser and dU-Tyr (structures shown in Fig. 1a), in our photolithographic microarray synthesis routine.^49^ We first characterized phosphoramidite coupling efficiency and the UV light dose required for complete removal of the photosensitive 2-(2-nitrophenyl)propoxycarbonyl (NPPOC) group. A 15 s coupling time for dU-Tyr, our standard coupling time for unmodified DNA amidites, leads to incomplete incorporation, but increasing the coupling time to 60 s improves the coupling efficiency from ∼95.7% (15 s) to ∼99.6% for dU-Tyr and from ∼99.2% to >99.9% for dU-Ser (Fig. S1). The efficiency of NPPOC photolysis was assessed in a terminal labelling approach by exposing the protected nucleotide after coupling to a gradient of 365 nm UV light, followed by the addition of a fluorophore. A higher fluorescent signal corresponds to a better removal of the photosensitive group. The results show that both C5-modified dU phosphoramidites require a higher UV light dose for efficient cleavage of the NPPOC group compared to unmodified dU (Fig. S2).

### Hybridization assays

After having identified the proper experimental conditions, we explored the impact of the base modification on hybridization. We synthesized homopolymers of dU, dT, dU-Ser and dU-Tyr (Fig. 1b) of 1 to 18 nt in length and analyzed the fluorescence signal intensity detected upon hybridization to a complementary Cy3-labeled DNA and RNA probe. Independent of the probe, we did not detect hybridization to dU homopolymers of any length, while dT strands hybridized only to the complementary DNA but not the RNA probe. Homopolymer strands of both modified nucleotides showed well-detectable signals after hybridization with either DNA or RNA probe. The data, summarized in the plots in Fig. 1c and S3 for hybridization to DNA and RNA, respectively, verified our hypothesis that signal intensity increases with oligonucleotide length. Our observation that strands of modified nucleotides yield higher signal than dT is consistent with reports describing enhanced base stacking of nucleotides carrying a nucleobase modification at the C5 position.^50^ However, we were surprised to detect decent signal intensities even for hybridization to very short strands: for example, hybridization of a DNA probe to a 3mer of dU-Tyr yields an average signal of 23% relative to the corresponding 18mer, a threshold that dU-Ser crosses at the 9mer mark and dT at the 10mer mark. Duplex melting unveiled another interesting finding. After a wash in deionized water, the fluorescence signal dropped to background level for all investigated variants with both complementary probes, except in the scenario of dU-Tyr strands hybridized to DNA. This modified duplex exhibited a surprisingly high stability against melting, so that even extended treatment at 60 °C in deionized water could not fully dissociate the complex, leaving up to ∼20% of the initial signal on the surface (data shown in Fig. 1c).

**Fig. 2:**
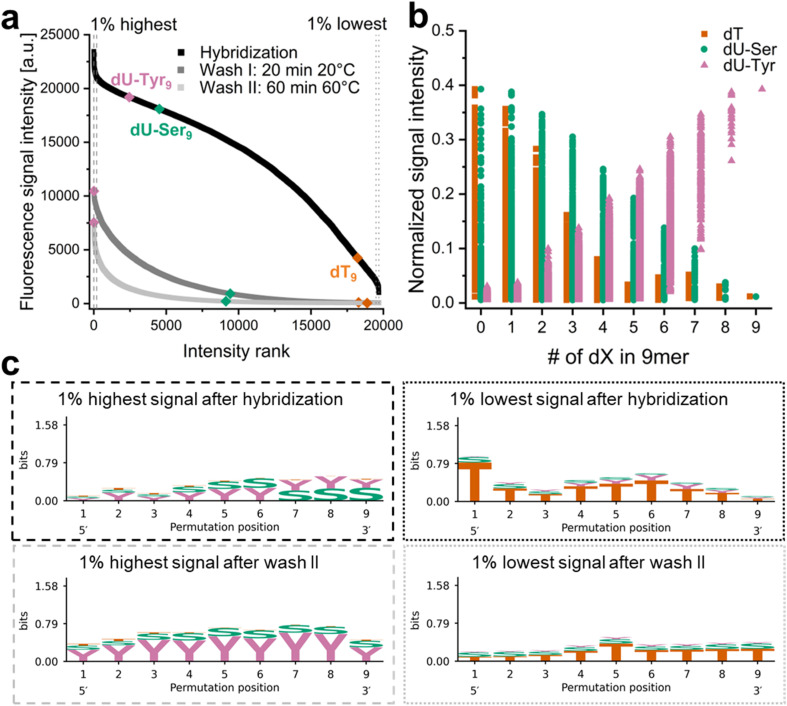
Hybridization to a 9mer permutation library of dT (T), dU-Ser (S) and dU-Tyr (Y) and melting assays. (a) All variants were ranked from highest to lowest fluorescence signal detected after hybridization (black), washing in water first at room temperature (dark grey) and then for a prolonged time at 60 °C (light grey). The positions of 9mers composed of only a single variant along each curve are highlighted in colour (dT = orange, dU-Ser = green, dU-Tyr = pink), sections of 1% highest and lowest signal intensity are indicated by dashed and dotted lines, respectively. (b) Illustration of the impact of the number of dT, dU-Ser or dU-Tyr within a variant on the remaining signal intensity after a stringent wash (fluorescence signal after wash #2 normalized to signal after hybridization). (c) Sequence logos of variants with 1% highest (dashed line) and lowest (dotted line) signal intensity after hybridization (black frame) and after the final wash (light grey frame).

In order to confirm that the detected fluorescent signal in our hybridization experiments is a result of base complementarity between the labeled probe and oligonucleotides on the surface, we synthesized a microarray with the previously discussed 1–18mer strands of dU/dT/dU-Ser or dU-Tyr together with a mixed-base 25mer, including variants in which all six dT in the sequence were substituted by either dU, dU-Ser or dU-Tyr. Hybridization with a complementary Cy3-labeled 25-nt probe yielded high signals for all four variants of the 25mer, as shown in the scans in Fig. S4a. In contrast, only background fluorescence was detected for the other spots on the array, indicating no cross-hybridization between the homopolymers and the 25mer mixed-base complement. These results indicate that the mere presence of the Tyr modification does not initiate nonspecific interactions with a fluorescently labeled non-complementary DNA probe that would give rise to elevated signal intensities compared to other dU variants. We were then interested in the behavior of oligonucleotides composed of combinations of the dU derivatives in hybridization assays. We focused on combinations of dT/dU-Ser/dU-Tyr but excluded dU polymers due to their poor hybridization profile, and synthesized the full permutation library of 9mers (3^9^ = 19 683 variants). We hybridized the DNA probe to this array and confirmed the overall pattern observed in the previous experiments: the distribution of signal intensities (Fig. 2a, data in black) shows that the 9mers are ranked corresponding to the previously shown data (dU-Tyr > dU-Ser ≫ dT). Applying harsh washing conditions (data in light grey), the duplex stabilizing effect of dU-Tyr compared to dU-Ser becomes once again evident, indicated by the position of the dU-Tyr 9mer moving to the first rank (highest signal intensity), but the dU-Ser 9mer is shifted to a more mid-range position. Fig. 2b illustrates the impact of the composition of the 9mers on the fluorescence signal that is still detectable after washing the array in hot water. Only ∼1% of the initial signal intensity is left for both the dU-Ser and dT 9mer. However, almost 40% of the fluorescence remains for the dU-Tyr 9mer. Overall, dU-Tyr content correlates with high leftover signal, but looking at the modification patterns of the variants yielding the top and bottom 1% of intensities (Fig. 2c) reveals that the mere presence of dU-Tyr does not automatically give rise to high fluorescence signal. Indeed, high signal patterns upon hybridization include both dU-Ser and dU-Tyr units, while the 5′ end of the 9mer appears to be less sensitive to modification (top left). A stringent wash changes this pattern (bottom left), as variants still yielding high fluorescence signals now show a clear preference for dU-Tyr at all positions, in line with our previous wash-off assays. Within the set of sequence variants yielding the lowest fluorescence, dT is prevalent at almost all 9 positions (top right). Washing attenuates the strong prevalence of dT in this range (bottom right). However, given that the remaining fluorescence signal after the final wash is very low for the majority of the dataset (light grey curve in Fig. 2a), rankings in the low intensity range should be taken with caution.

### Nucleobase-modifications in aptamer binding

Next, we aimed to investigate the effect of the incorporation of dU-Ser and dU-Tyr in DNA aptamers. In contrast to our previous experience with studying protein binding on microarrays in a multistep approach,^33,51^ we wanted to explore the feasibility of a single-step assay using a fluorescently labelled protein as target. In preliminary experiments on streptavidin aptamers^48,52^ synthesized by microarray photolithography, we identified conditions that yield satisfactory signal-to-noise and signal-to-background ratios with Cy3-labelled streptavidin. These performance metrics were determined by comparing the median signal intensity for the unmodified aptamer, *e.g.* St-2-1, with that of a sequence of identical length and nucleotide composition (St-2-1_rev), and with features that contain only the linker (bkg), respectively (example shown in Fig. S5), indicating that the detected fluorescence can be ascribed to specific binding.

#### Aptamer St-2-1

**Fig. 3:**
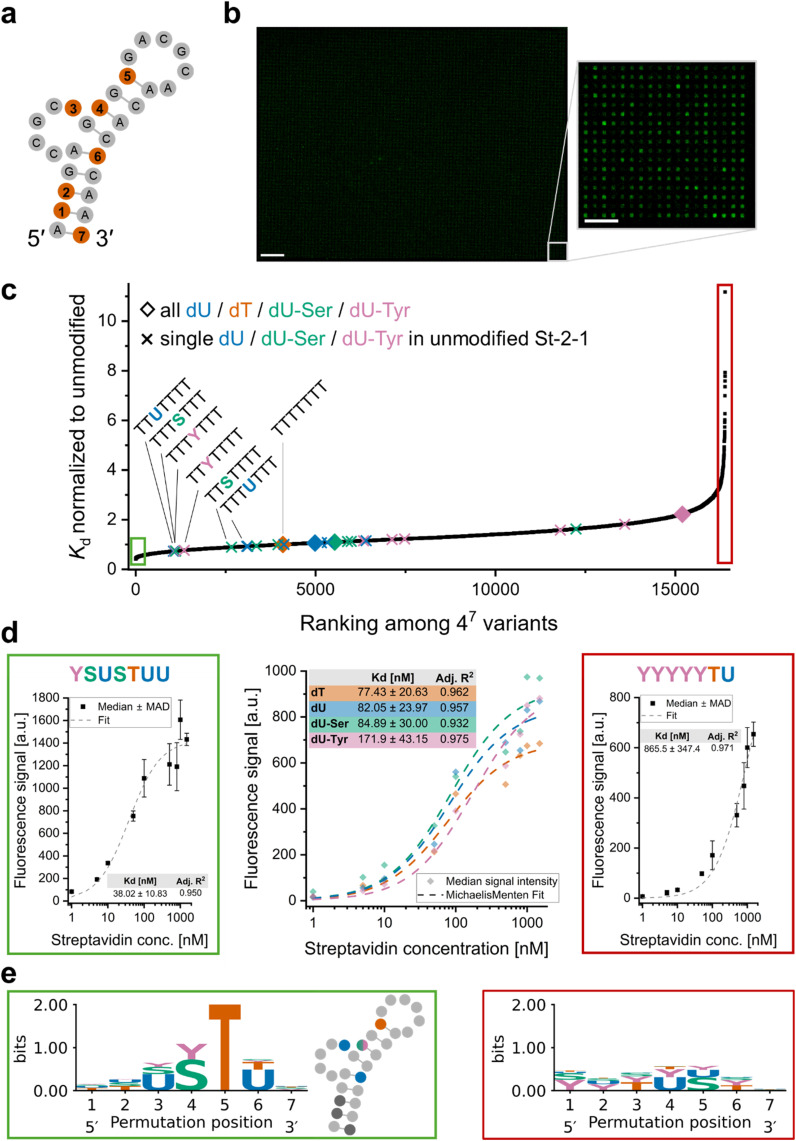
Site-specific permutation of the streptavidin-targeting DNA aptamer St-2-1. (a) Aptamer structure as predicted by Bing *et al.*^48^ with the seven positions selected for permutation (positions containing a dT in the published sequence) indicated in orange. (b) Representative scan recorded upon incubation of the entire permutation library with 16 384 variants with 1 nM Cy3-labelled streptavidin. Left: Full synthesis area (scale bar = 500 µm), right: excerpt of ∼0.2% of the microarray surface (scale bar = 100 µm, size of individual spots: 14 × 14 µm). (c) *K*_d_ values determined for all 4^7^ sequences normalized to the St-2-1 wildtype and ranked from smallest to largest. Diamonds indicate variants containing only a single type of nucleotide at all interrogated positions (corresponding to data in centre above), “×” indicates sequence variants with only a single dT replaced by dU, dU-Ser or dU-Tyr, with labels indicating all those carrying the substitution at position 3 or 4. (d) Example plots of fluorescence intensities detected at 1 nM to 1.5 µM streptavidin concentration and fit to estimate *K*_d_ values of selected candidates in the library (green frame: data for variant with lowest *K*_d_, red frame: highest *K*_d_, centre: data for variants with all permuted positions 1–7 carrying the same type of dU derivative – wild-type in orange, all positions dU in blue, dU-Ser in green and dU-Tyr in pink). (e) Sequence logos illustrating the frequency of each nucleotide at each site of the permutation within the set of 82 variants (∼0.5% of dataset) with lowest (top, green frame, including an insert showing position of relevant dU variants in the aptamer structure) and highest *K*_d_ (bottom, red frame).

St-2-1 had served as reference in previous studies, is showing nanomolar affinity to streptavidin,^48,51–53^ and is sufficiently short (29 nt) to ensure good quality in chemical synthesis. We therefore decided to substitute all seven dT nucleotides in its sequence with either dU, dT, dU-Ser or dU-Tyr (Fig. 3a), yielding a permutation library with 16 384 variants. The microarray format allows us to assess the binding interactions of the entire library in a single experiment and to directly compare all variants with each other (example scan in Fig. 3b). Incubation with Cy3-labelled streptavidin did not indicate any correlation of the number of a certain type of modification with fluorescence signal as a measure for target binding (compare Fig. S6). This result suggests that certain combinations of dU derivatives within the aptamer structure enhance binding rather than the mere presence of a distinct functional moiety attracting the protein in an unspecific manner. In order to detect differences in binding affinity, the library was incubated with increasing concentrations of streptavidin, and the fluorescence intensity data were fit to a hyperbolic regression model to estimate a *K*_d_ value for each variant. Ranking the variants according to affinity (Fig. 3c) yields a broad range of *K*_d_ values, with the majority of combinations shown to bind streptavidin with lower affinity than the unmodified aptamer (datapoint indicated in orange at *y* = 1).

However, several thousands of combinations appear to have higher target affinity than the wild-type. For single modifications (datapoints marked with “×” in Fig. 3c), we find that any variant with either dU, dU-Ser or dU-Tyr at position 3 or 4 rank better than the unmodified St-2-1. The *K*_d_ obtained for the unmodified St-2-1 (data shown in orange in the central plot in Fig. 3d) is consistent with previously reported values that range from approximately 40 nM ^48^ to 100 nM.^53^ Substituting all seven dT with either dU or dU-Ser has only a minor impact on affinity, but replacing all dT with dU-Tyr significantly affects protein binding. We examined the extremes of the affinity distribution (representative examples shown in Fig. 3d, highlighted in green and red, respectively) and searched for patterns in the sequence variants yielding the 82 lowest *K*_d_ values (top 0.5% of the entire dataset). This analysis revealed a site-specific combination of modifications that promote target binding. Illustrated in the sequence logo (Fig. 3e), target affinity appears to benefit from the presence of a dU at positions 3 and 6, whereas dU-Ser and dU-Tyr promote target binding when introduced at position 4. The data show the particular sensitivity of position 5, as this position appears conserved with dT units well beyond the top 0.5% range: of the 1638 sequences with highest affinity (top 10% of dataset), ∼80% of variants have dT at position 5. In contrast, when looking at the very other end of the ranking, dT is found at position 5 in only ∼1% of the bottom 10% of binding affinity. The requirement for dG as the first nucleotide in the loop to enable efficient target binding has been described previously,^48^ but based on our data we also suspect the neighboring dT at position 5 to be interacting with streptavidin. Furthermore, the absence of dU in the top candidates suggests that contact of the methyl group at C5 of dT with the protein is important. While position 7 at the 3′ terminus appears indifferent to introduction of any modification, positions 1 and 2 close to the 5′ terminus show a tendency to disfavor dU-Ser and dU-Tyr. A potentially stabilizing effect of these modifications on the duplex in the stem, as suggested in hybridization experiments (Fig. 1c), does not improve target binding. This observation is in line with previous reports on the marginal impact on *K*_d_ upon the introduction of LNA nucleotides – known to increase duplex melting temperature – to the stem region of St-2-1.^53^

#### Aptamer St-23-2

**Fig. 4:**
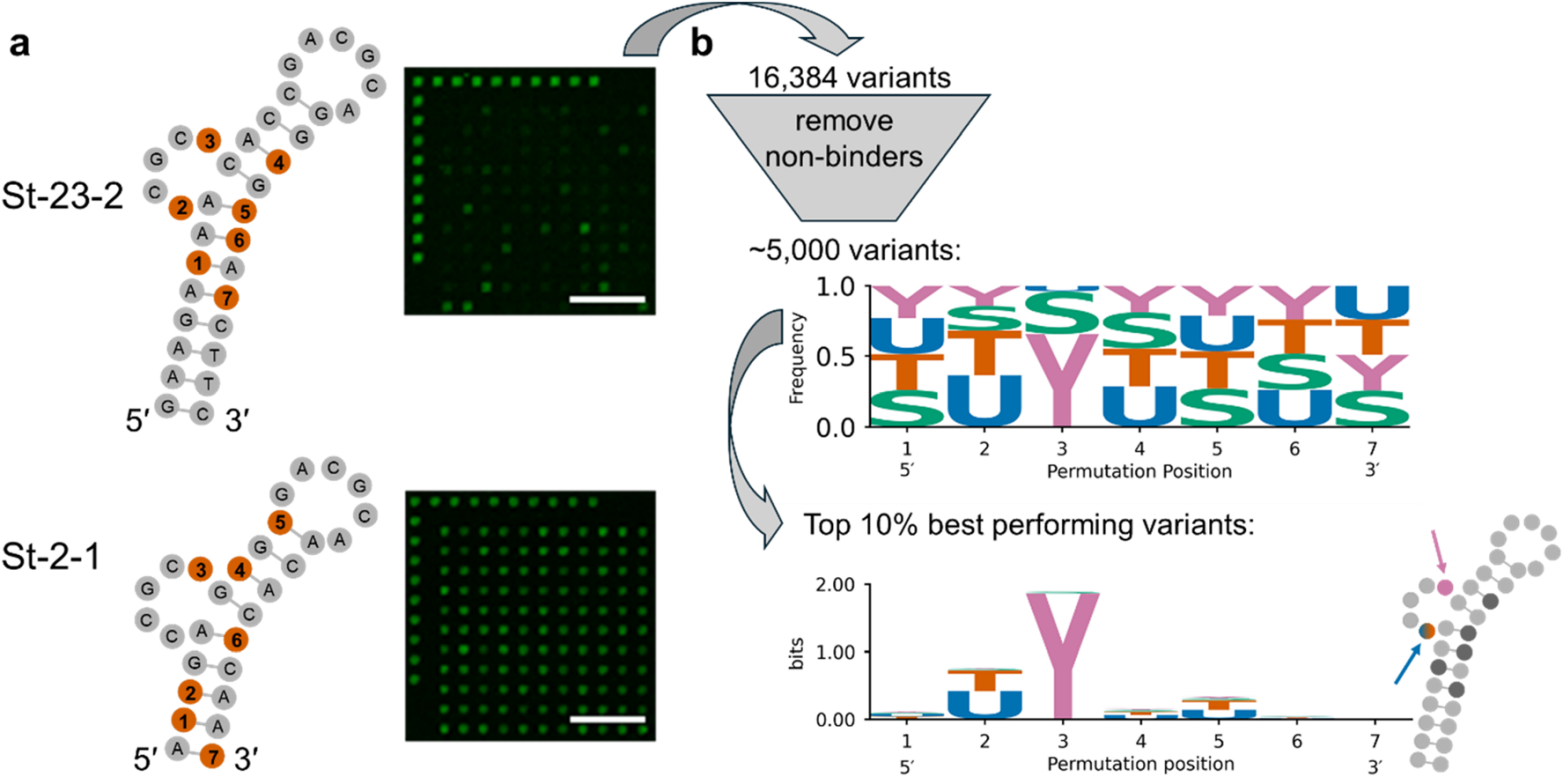
Assessing the impact of nucleobase-modified nucleotides in the St-23-2 aptamer. (a) Comparison of predicted secondary structures of the two investigated aptamers, with the seven dT that were substituted with dU, dU-Ser or dU-Tyr shown in orange, and excerpts of microarray scans recorded upon incubation of the permutation libraries with 1500 nM Cy3-labelled streptavidin (scale bar = 100 µm). (b) The data was filtered to retain only variants of St-23-2 with fluorescence signal significantly above background level in the dataset, which was then analyzed for the frequency of each nucleotide at each permuted position within the remaining dataset (frequency logo, above) and to find sequence patterns that appear to promote protein binding (sequence logo of the 10% top candidates and insert highlighting relevant positions within the proposed secondary structure, below).

Aptamer St-23-2 was identified by Bing *et al.*^48^ with a predicted secondary structure resembling St-2-1 but with an 8 bp long stem instead of 5 bp in St-2-1 (see Fig. 4a). It shares the loop and bulge sequence with St-2-1, except for the first dT residue in the bulge, and was described to have comparable affinity to streptavidin. In our hands, the results for protein binding deviate significantly from those for St-2-1 when directly comparing both aptamers on the same microarray, as the fluorescence signal after incubation with Cy3-labelled streptavidin barely exceeded background level for the unmodified St-23-2 (shown in Fig. S5). Nevertheless, the St-23-2 aptamer fared better once we started introducing modifications as we detected an up to ∼6-fold increase in signal intensity by substituting some dT with dU derivatives. In order to investigate the impact of these substitutions in a more systematic way, we synthesized a permutation library consisting of any combination of either dT, dU, dU-Ser or dU-Tyr at seven selected positions in the aptamer sequence (16 384 combinations) and incubated the corresponding array with increasing concentrations of Cy3-labelled streptavidin. As expected from our preliminary tests, we observed many poor binders, evidenced by comparing the scans in Fig. 4a. Most variants did not meet our threshold criteria for the estimation of *K*_d_ values (fluorescence signal above 10× SD of the background intensity and *r*^2^ of fit >0.7) and were therefore excluded from the analysis, including the wild-type St-23-2. The residual dataset contained only ∼30% of the full library with *K*_d_ values fluctuating in the µM range. Investigating the remaining sequences revealed the significant impact of a modification placed at position 3, as shown in the frequency logo in Fig. 4b and, at the same time, the almost complete absence (<0.5%) of dT at this position. In fact, more than 90% of all variants of the 16 384-member library that contain dU-Tyr at position 3 are present in the final dataset. In order to discover sequence patterns that can be associated with efficient protein binding both in terms of affinity and detectability, we ranked the variants in the residual dataset according to a scaled *K*_d_ (estimated *K*_d_ divided by median signal intensity at maximum target concentration). When assessing the top 10% best performing variants, we found a distinct pattern that highlights – in addition to the requirement for dU-Tyr at position 3 – a preference for either dU or dT at position 2 (illustrated in the sequence logo of top 10% variants in Fig. 4b). We also included in our investigation a library containing 2048 (2^11^) variants where each nucleotide in both the bulge and the loop has been replaced with dU-Tyr. Only a handful of these variants appeared in the subset that meets the threshold criteria for binding (Fig. S7), all of them modified with dU-Tyr at the 3′-most position in the bulge (corresponding to position 3 in Fig. 4). This data suggests that the presence of an additional dU-Tyr at the second position in the loop or position 1 or 2 in the bulge does not disrupt protein binding, but replacing any other nucleotide in these segments has a detrimental effect on the interaction with streptavidin.

As seen with two streptavidin aptamers, we find that the effect of nucleobase modifications on protein binding is dependent on the exact position of the insertion. We observed that even in supposedly structurally similar aptamers for the same target, the insertion of non-canonical functional groups seems to affect target binding differently. Our observations underline the need for structural information to reveal the definite modes of interactions involved and ultimately understand the mechanism of target binding, which remains unclear at this point.

Nevertheless, microarrays can be seen as a complementary tool to well-established methods for the identification and refinement of aptamers, since microarrays deliver non-random, sequence and position-specific oligonucleotide variants through parallel chemical synthesis. In addition, these surface-bound libraries can rapidly provide a broad overview of sequence patterns as well as detect off-target and non-specific effects.

## Conclusions

Microarray synthesis *in situ* is based on phosphoramidite chemistry. It is therefore amenable to the same chemical complexity that solid-phase synthesis of oligonucleotides can offer. This work falls in line with our aim to enrich the chemical toolbox compatible with photolithography and presents the incorporation of two nucleobase-modified building blocks. We focused on nucleobase analogs decorated with the side chain of the amino acids serine and tyrosine (dU-Ser and dU-Tyr, respectively), whose potential to expand the molecular functionality of DNA has been studied extensively in SELEX assays.^16,17,23^ While being a powerful method to select for high-affinity binders from a very large set of different molecules, meaningful information on sequence patterns of variants binding with lower affinity but higher specificity – potentially causing off-target effects in downstream applications – can get lost during selection. In the context of DNA modifications, the accessible combinatorial space is limited by enzymatic promiscuity. Microarrays, on the other hand, can assay every member of a nucleic acid library simultaneously and individually regardless of its nature or chemical composition. This allowed us to directly compare oligonucleotides containing either dT, dU, dU-Ser or dU-Tyr and permutations thereof in hybridization and protein binding assays. In our experiments, we observed that a local accumulation of base-modified nucleotides significantly improved hybridization to complementary probes, indicating a greatly increased duplex stability with dU-Tyr modified oligonucleotides. Our results show enhanced hybridization performance with increasing size of the functional group at the C5 position (dU < dT < dU-Ser < dU-Tyr), suggesting improved base stacking.^50,54^ In particular, oligonucleotides containing consecutive dU-Tyr moieties displayed a duplex stability that is highly unusual for short polyuridylates, appearing strikingly resistant to washes with deionized water. We also synthesized dT-permutation libraries of two known aptamers targeting streptavidin to assess the effect of the modified nucleotides on protein binding. The results highlight the impact of the exact positioning of modified nucleotides in aptamer sequences: in the St-2-1 aptamer, we identified a position that does not tolerate any variant other than dT for high-affinity binding to streptavidin and, in contrast, substitution of dT with other dU derivatives at three distinct sites promotes protein binding. Our investigation on the aptamer St-23-2 can be considered a prime example of the impact of nucleobase modifications, since it showed how the presence of a single modified nucleobase at a specific position enables the detection of a binding signal which is absent with the wild-type aptamer. These results are in line with previous reports describing the power of the introduction of noncanonical functional groups to improve the affinity of oligonucleotides for various targets.^10,18,25,26,28^ Such complex surface-bound sequence libraries, which can be used to investigate the compounding effects of various modifications simultaneously and at high-throughput, can be seen as a promising addition to the existing toolset and methods that aim to discover and identify candidate aptamers in diagnostic applications like biosensors.^6^ At the same time, we also regard nucleic acid arrays as platforms to study the impact of nucleobase modifications on protein recognition. Moreover, considering that nucleobase modifications have potential to improve the therapeutic profile of antisense oligonucleotides and nucleic acid vaccines,^55^ our parallel synthesis approach could potentially facilitate the process of initial screening for therapeutically relevant interactions, including the identification of off-target effects.

Our comparison of two aptamers with very similar predicted structures shows that the impact of nucleobase modification and position on target binding needs to be individually assessed for each aptamer. Further work should aim at shedding light on the molecular mechanisms underlying the improved binding affinity of these chemically modified aptamer variants using appropriate analytical and biophysical methods. This study continues to underline the value of microarrays in aptamer research, positioning them as tools complementary to established methods for the identification and improvement of aptamers. However, while this report shows our ability to generate large oligonucleotide libraries with nucleobase modifications inserted at specified positions and apply them in protein-binding assays, microarray assays are inherently limited by the need for a fluorophore for detection. Though some examples have shown that fluorescence assays with surface-bound nucleic acids are able to approximate protein binding in solution,^33,37,56,57^ independent validation of aptamer candidates by complementary methods will be required to get a definite answer on binding characteristics.

## Author contributions

E. S. conceived and performed the experiments, analyzed the data and wrote the original draft of the publication. J. L. acquired funding and supervised the project. Both authors reviewed the manuscript and agreed to its content.

## Conflicts of interest

There are no conflicts to declare.

## Supplementary Material

RA-016-D6RA01942K-s001

RA-016-D6RA01942K-s002

## Data Availability

The data supporting this article have been included as part of the supplementary information (SI). Supplementary information: additional data plots for the evaluation of the base-modified phosphoramidites, hybridization and melting of a complementary RNA and a mixed-base 25mer probe, and supplementary results for aptamer-target incubation. Fluorescence intensity data for all experiments. See DOI: https://doi.org/10.1039/d6ra01942k.
