# Supplementary material for: Combinatorial and rational synthesis of complex, base-modified aptamer libraries on microarrays
Source: RSC Adv. 2026 May 19;16(29):26976–87. doi: 10.1039/d6ra01942k (PMC13187762; doi:10.1039/d6ra01942k)
Supplement: RA-016-D6RA01942K-s002 [file RA-016-D6RA01942K-s002.pdf]

Supplementary Information (SI) for RSC Advances.
This journal is © The Royal Society of Chemistry 2026

Supporting Information

Combinatorial and rational synthesis of complex, base-modified aptamer libraries on microarrays

Erika Schaudy[a], Jory Lietard[a]

[a]Institute of Inorganic Chemistry, University of Vienna, Vienna 1090, Austria

Table of Contents

Determination of coupling efficiency 2
Determination of photolysis efficiency 2
RNA probe hybridization and melting 3
25mer probe hybridization 4
Aptamer target binding comparison 5
Modifications in St-2-1 6
dU-Tyr in bulge and loop of St-23-2 7

Determination of coupling efficiency

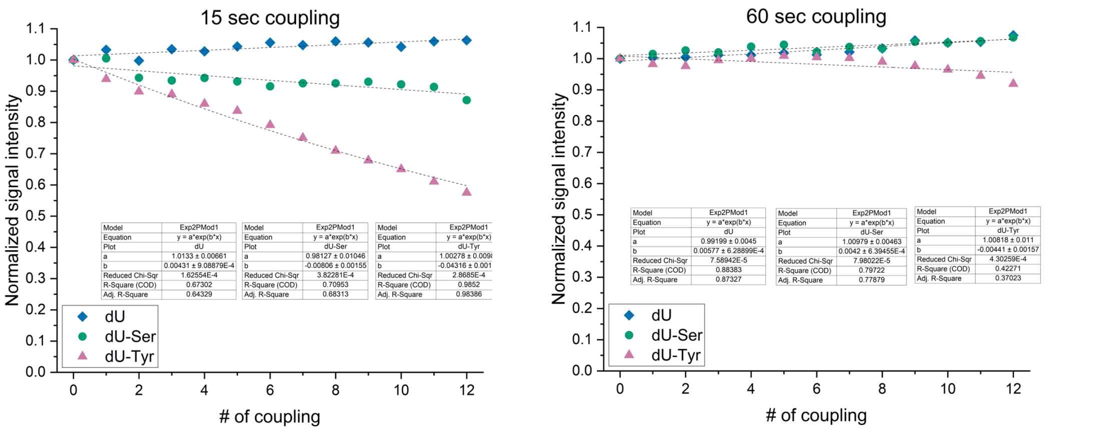

Figure S1. Impact of coupling time on the stepwise coupling efficiency of dU, dU-Ser and dU-Tyr phosphoramidites, comparing a reaction time of 15 seconds (left) and 60 seconds (right).

Determination of photolysis efficiency

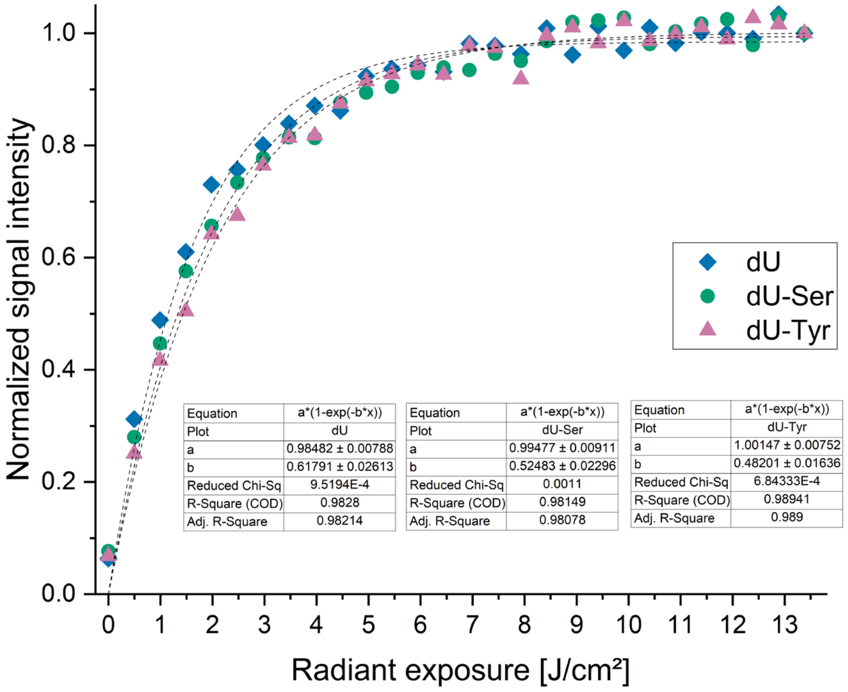

Figure S2. Evaluation of photolysis efficiency based on terminal labelling. An exponential model function ($y = a * (1-e^{-bx})$) was fit to the data to estimate the light dose required for 95% photodeprotection of the NPPOC-protected phosphoramidites (~5.4 J/cm² for dU, ~5.9 J/cm² for dU-Ser, ~6.2 J/cm² for dU-Tyr).

RNA probe hybridization and melting

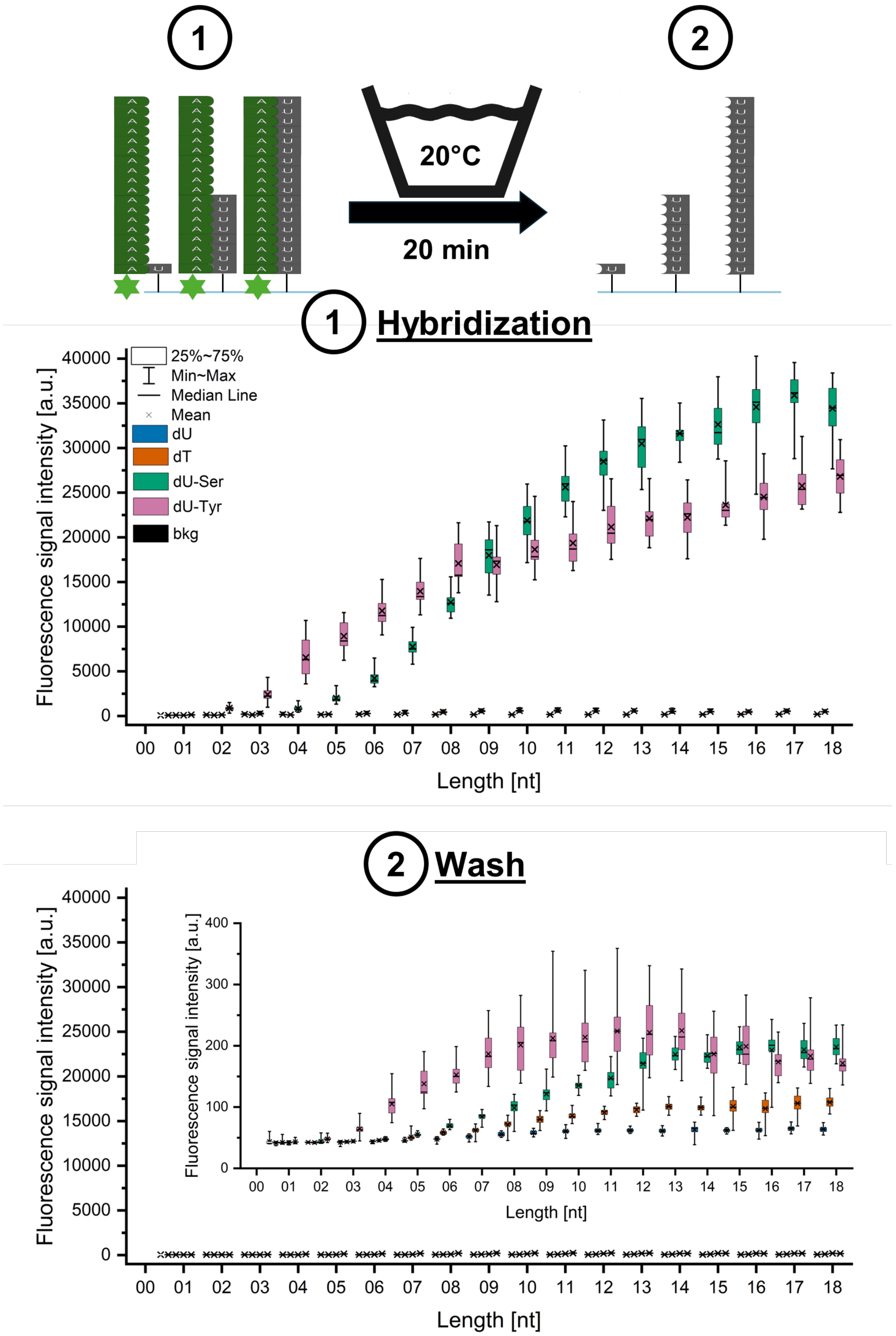

Figure S3. Data and schematic representation for (1) hybridization with a complementary Cy3-labeled RNA probe to a microarray with 1-18mers of dU, dT, dU-Ser and dU-Tyr, followed by 20 min wash with water at room temperature (2), including an insert with adjusted y axis to illustrate minor differences in signal intensities close to background level.

25mer probe hybridization

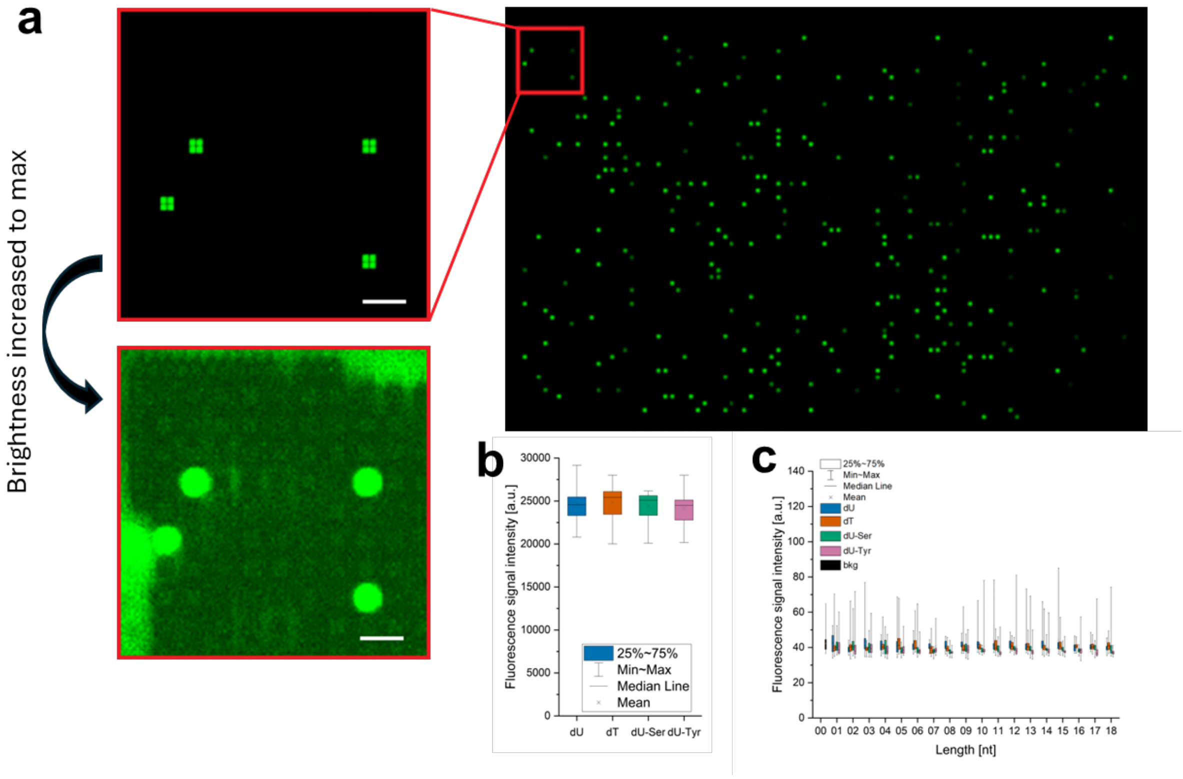

Figure S4. Hybridization with a Cy3-labeled mixed-nucleotide 25mer DNA probe to a microarray with complementary sequences (complementary base to all A in probe either U, T, U-Ser or U-Tyr) and 1-18 nt long homopolymer strands of dU/dT/dU-Ser/dU-Tyr. a) The scan and its excerpt (~1.5% of microarray, scale bar = 100 µm) of the microarray after hybridization, with extracted signal intensity data for the 25mer (b) and the 1-18mers (c) highlight both successful hybridization to the surface-bound complements independent of the dU variant present in the 25mer, and the absence of any nonspecific binding interactions with C5-modified nucleotide strands of different length.

Aptamer target binding comparison

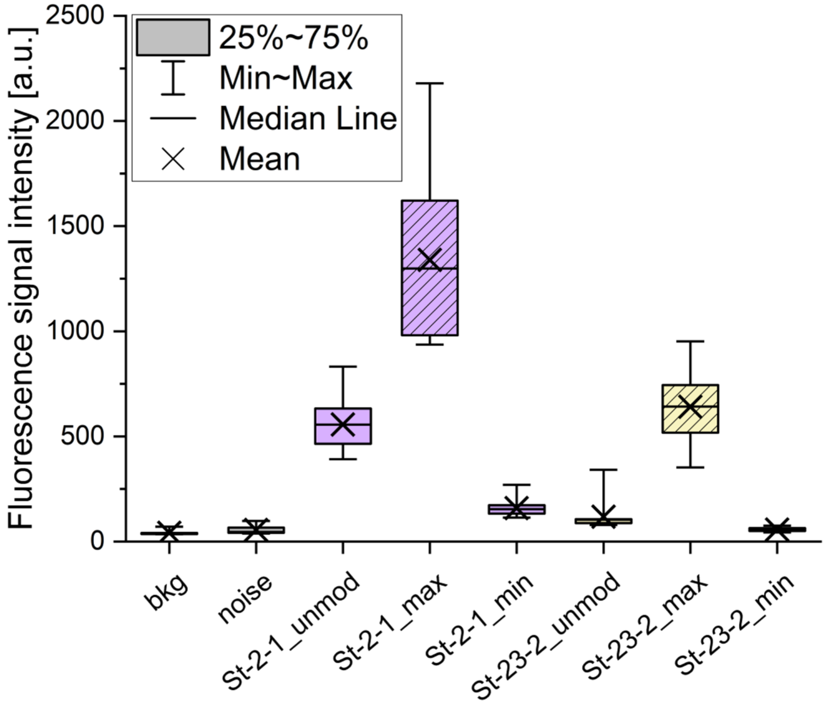

Name	Pattern of dT substitutions
St-2-1_unmod	TTTTTTT
St-2-1_min	UUUUUUU
St-2-1_max	TTYTYYT
St-23-2_unmod	TTTTTTT
St-23-2_min	TYTTYTT
St-23-2_max	YUYUTTY

Figure S5. Direct comparison of the two streptavidin aptamers St-2-1 and St-23-2 with their sequences as published ("unmod") and with a selected set of combinations of dU derivatives highlights the base modification's impact on target binding, as indicated by a substantial change in detected fluorescence signal (data shown for sequence variants yielding highest – "max" – and lowest – "min" – signal). Signal-to-noise ratio is ~12, signal-to-background ratio ~14, based on the comparison with unmodified St-2-1.

Modifications in St-2-1

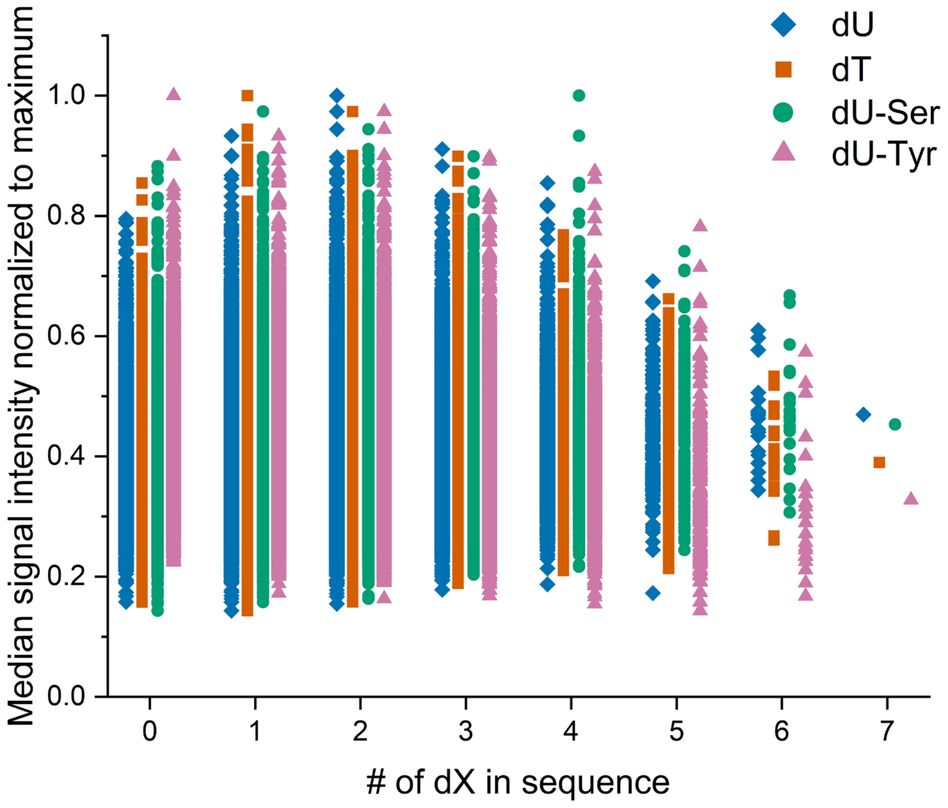

Figure S6. Relation of number of substitutions within the St-2-1 aptamer sequence (substitution of seven dT in the unmodified sequence with either dU, dU-Ser or dU-Tyr, yielding a permutation library of 16,384 variants) and the median fluorescence signal intensity for aptamer binding when applying 100 nM Cy3-labeled streptavidin.

dU-Tyr in bulge and loop of St-23-2

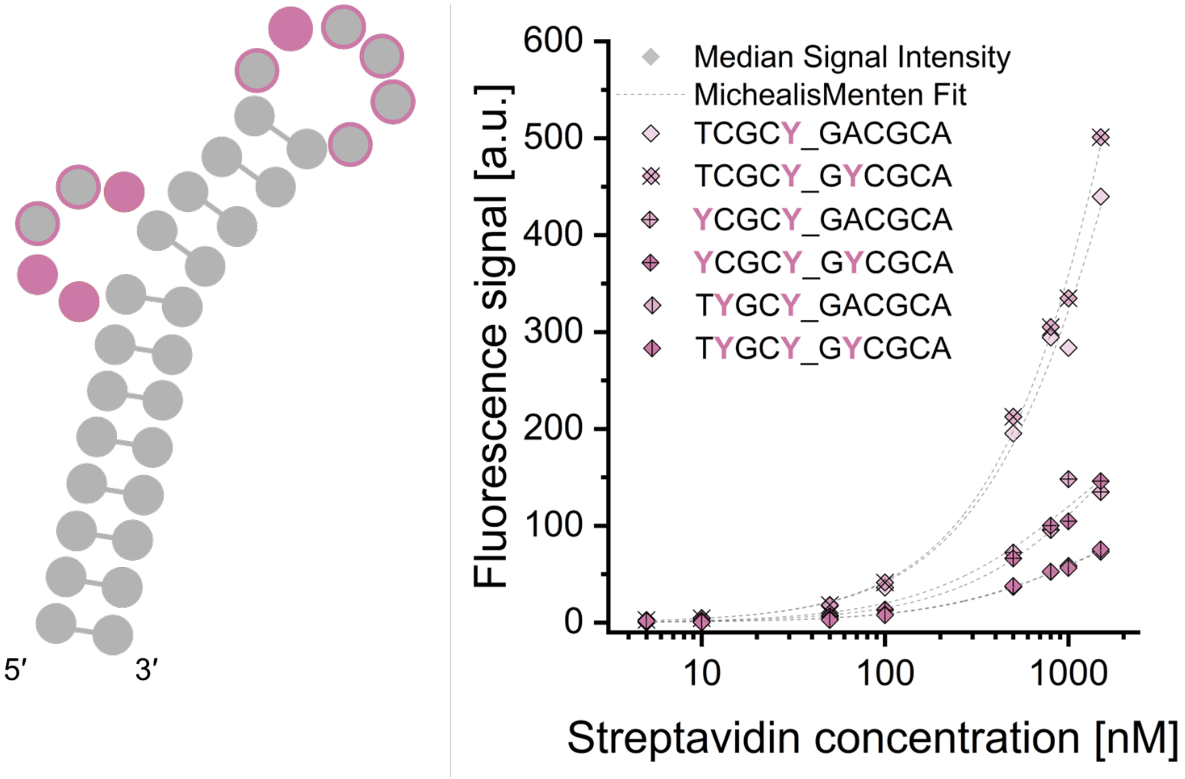

Figure S7. Assessment of dU-Tyr substitutions in the bulge and loop section of aptamer St-23-2. Left: Schematic representation of the predicted secondary structure with positions that have been permuted to dU-Tyr framed in pink, and those positions at which the introduction of dU-Tyr enabled protein binding filled with pink. Right: Fluorescence signal for binding of fluorescently labelled streptavidin at different concentrations to different variants of St-23-2 with site-specifically introduced dU-Tyr and hyperbolic fit to estimate binding affinity.
